# Managing xylazine-involved overdoses in a community harm reduction setting: lessons from Tijuana, Mexico

**DOI:** 10.1186/s12954-024-01143-2

**Published:** 2025-01-04

**Authors:** Lilia Pacheco Bufanda, Alejando González Montoya, Brenda Torres Carrillo, Mariana Alejandra Gonzalez Tejeda, Luis A. Segovia, Alhelí Calderón-Villarreal, Joseph R. Friedman

**Affiliations:** 1 Prevencasa A.C. Harm Reduction Clinic, Tijuana, Baja California Mexico; 2 Xochicalco University, Tijuana, Baja California Mexico; 3 Public Health Department, California State University, San Marcos, San Marcos, CA USA; 4 Asociación Bajacaliforniana de Salud Pública A.C, Tijuana, Baja California Mexico; 5 Department of Psychiatry, University of California, San Diego, San Diego, CA United States

## Abstract

**Background:**

Xylazine is a α2-adrenergic receptor agonist, used for sedation in veterinary contexts. Although it is increasingly found in overdose deaths across North America, the clinical management of xylazine-involved overdoses has not been extensively studied, especially in community-based harm reduction settings. Here we present a clinical series of xylazine-involved overdose and share the clinical approach and lessons learned by a community overdose response team in Tijuana, Mexico amidst the arrival of xylazine.

**Case Presentation:**

We present three cases describing the clinical management of patients with xylazine-involved overdoses that occurred in close proximity to the Prevencasa community harm reduction clinic. The long period of post-naloxone sedation in xylazine overdoses is a unique clinical feature. The first case is a 61-year-old man with longstanding opioid and methamphetamine use disorder found hypoxic, who received 4.0 mg of intranasal naloxone, and quickly began respirating well. He remained unconscious for 20 min, and upon awakening, experienced withdrawal symptoms, agitation and confusion, and exposed himself to considerable physical danger by entering a local roadway. The second is a 28-year-old man who primarily uses stimulants, who overdosed while trying “China White”. His oxygen saturation improved from 81 to 100% with supplemental oxygen and field management, and he did not require naloxone administration. He recovered consciousness after 40 min. The third patient is a 36-year-old male who was found down, saturating at 20%, who received 0.4 mg intramuscular naloxone, was placed in recovery position, and remained unconscious for 12 min before making a complete recovery. The first and third patients provided urine and drug samples that tested positive for xylazine and fentanyl.

**Conclusions:**

We describe insights about the clinical management of combined xylazine-fentanyl involved overdoses in the field, from a community harm reduction context where xylazine arrived suddenly spurring a large number of overdoses. In response to the long period of post-naloxone sedation inherent to xylazine overdoses, the clinical team learned to center oxygenation—not consciousness—as the key parameter of interest for the titration of naloxone, increase emphasis on field airway management, portable oxygen administration, and scene safety, and employ xylazine testing strips for urine and direct substance analysis to educate the patient population about health risks.

## Background

Xylazine—often referred to as “tranq” or “*anastesia de caballo*” in Spanish—is a α2-adrenergic receptor agonist, used for sedation in veterinary contexts [1–3]. Unlike other α2 agonists, such as clonidine or dexmedetomidine, xylazine has not been approved for human consumption in the United States (US) or other jurisdictions [4]. Xylazine appeared in the illicit drug market of Puerto Rico in the early 2000s where it was used to augment the effects of heroin [5]. It subsequently emerged as an additive to illicit fentanyl in mainland US in the late 2010s and early 2020s [2, 6]. The prevalence of xylazine among fatal overdoses has risen exponentially in subsequent years [7–9], however its causal role in overdose events remains a matter of scientific discussion [10]. Qualitative data suggest that xylazine may increase the complexity and difficulty of life-saving treatment during overdoses [2].

The arrival of xylazine-fentanyl co-use to the North American overdose crisis has been highly notable, and xylazine has been identified as an emerging threat by the government of the US [1], Chile [11], by the Organization of American States [12], and more recently by Mexico [13]. However, the scope of the problem in the Mexican context deserves further study, as robust epidemiological data are not yet available. Preliminary evidence suggest that xylazine may be found in high concentration among fentanyl samples in Tijuana [14]. Additionally, little information is available in the literature about the clinical management of xylazine-involved overdoses, especially in community harm reduction spaces. Here we present a series of clinical cases of xylazine-fentanyl overdose, which exemplify the clinical approach developed, and the lessons learned, by an overdose response team at Prevencasa, a community harm reduction clinic in Tijuana, Baja California, Mexico. The patients whose cases are featured here provided written consent.

Prevencasa serves a large population of people who inject and smoke illicit opioids, many of whom have been deported from the US. The clinic has more than a decade of experience responding to overdose events in the surrounding neighborhood of *Zona Norte* (which concentrates a large number of illicit drug sales points and open-air drug use). Although overdose events were initially sporadic, they became increasingly common with the arrival of illicit fentanyl to Tijuana [15]. In response, the clinic trained an overdose response team, with designated clinical members (usually one physician, one nurse, and one or more harm reduction staff members) equipped with naloxone and other supplies. All team members were trained in how to assess for the clinical signs and symptoms of opioid overdose, as well as how to administer naloxone administration. MD and RN team members were additionally trained in how to rapidly assess key vital signs, especially using pulse oximetry, and in the administration of oxygen and other life-saving measures.

### Case presentation 1

**Fig. 1:**
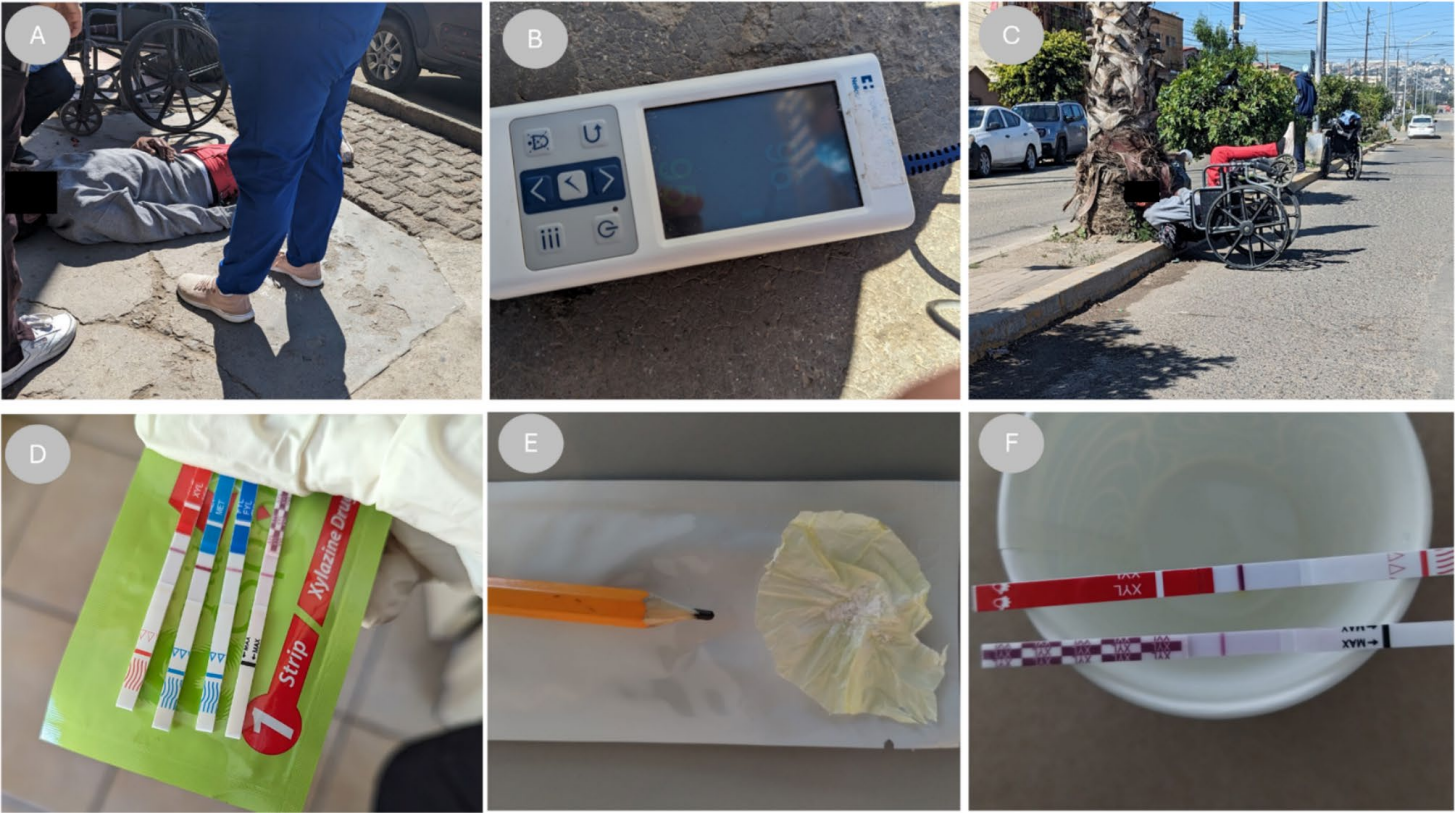
Photos From Overdose Response Team – Cases Involving Xylazine. **A**) Overdose response, **B**) pulse oximetry post naloxone administration, **C**) physical danger consequent to delirious state following return-to-consciousness **D**) Urine results showing xylazine, fentanyl, methamphetamine positivity, **E**) sample of “China White” tested for xylazine **F**) xylazine positivity for China White sample

A 61-year-old man with opioid and methamphetamine use disorder, well-known to the clinic, who uses a wheelchair due to a complete right lower limb amputation, was found lying face first on the ground, across the street from the harm reduction clinic, bleeding from a head laceration (Fig. 1). His respiratory rate was imperceptible, he was cyanotic, and his pupils were pinpoint. A nurse administered a 4.0 mg intranasal dose of naloxone, and the patient quickly resumed visibly respirating, however, he did not regain consciousness. Pulse oximetry showed the patient saturating at 96%, with a pulse of 95 bpm, and auscultation revealed normal breath sounds. The patient remained unarousable for approximately 20 min, during which time the clinical team monitored his oxygenation status, breath sounds, and pulses. He subsequently awoke suddenly in a delirious state and became agitated, endorsing distressing withdrawal symptoms. He pulled himself into his wheelchair and wheeled himself into traffic in a nearby roadway, exposing himself to considerable physical risk. There he once again lost consciousness, falling backwards onto the curb. Several community members placed him back into his wheelchair, and brought him into the clinic, where he accepted post-overdose monitoring. His vitals remained stable for the next two hours. Urine screening revealed positivity for xylazine, fentanyl, and methamphetamine. The patient reported a two-decade history of opioid use disorder, and endorsed currently both smoking and injecting ‘China White’ (a fentanyl powder formulation that increasingly contains xylazine in Tijuana) mixed with methamphetamine every two hours. The patient provided a drug sample to the on-site drug checking program, as well as a urine sample. Both tested positive for xylazine, using two different brands of immunoassay qualitative testing strips, as well as fentanyl. The patient received counseling about the risks of xylazine and fentanyl.

### Case presentation 2

A 28-year-old man with long-standing methamphetamine use disorder and no other known medical history was found unconscious, in a supine position, on the sidewalk, a few meters from the harm reduction clinic. Community members on scene reported that they observed him smoking a substance from a glass pipe prior to losing consciousness. He was not arousable, and pulse oximetry showed an oxygen saturation of 81% and a heart rate of 92 beats per minute. His blood pressure and blood glucose were within normal limits. He responded to verbal and physical stimuli only by opening his eyes but was not verbally responsive. On several occasions he attempted to sit up but was too sedated to do so. The overdose response team delivered oxygen through nasal cannula via a portable oxygenation concentrator. The patient’s oxygenation saturation immediately responded by rising to 100%. The patient was brought into the clinic for vital sign monitoring and supplemental oxygen. The response team opted against naloxone administration given that his vital signs stabilized with supplemental oxygen. After 40 min the patient awoke calmly, was alert and oriented to self, place, and date, and was informed about the process of his care, as well as the possible exposure to xylazine. He endorsed having used the street opioid mixture “China White” in a recreational fashion, although typically he only consumes stimulants, and it is unusual for him to consume opioids. He denied having used benzodiazepines, alcohol or other psychoactive substances. The patient declined urine or substance testing or any further medical care and left the clinic. He was seen one week later for follow-up care and was found in stable condition and received counseling.

### Case presentation 3

A 36-year-old man with long-standing opioid use disorder was found sitting on a curb, leaning back onto a car, very close to the harm reduction clinic, his eyes were covered by a hat. He was sitting among a group of various community members who were smoking and/or injecting illicit opioids. He did not respond to verbal cues, which prompted a member of the overdose response team to attempt to arouse him with touch, to which he also did not respond. The overdose response team member noted that the patient’s extremities were stiff, cold, and diaphoretic. The rest of the response team was summoned, the patient was laid flat on the ground, and was noted to be cyanotic, with blue lips and nails. Pulse oximetry revealed an oxygen saturation of 20%. A dose of intramuscular naloxone of 0.4 mg was administered, and within the next 2 min, oxygen saturation reached 99%, although the patient remained unresponsive to verbal and physical stimulus. The patient was placed on his side in recovery position, vital signs were assessed for the next 12 min, at which time he began to move his arms and legs in an agitated fashion, with his eyes remaining closed. Shortly thereafter he opened his eyes, was fully oriented, and was able to follow verbal guidance. He accepted post-overdose care in the clinic and offered a sample from the syringe containing the substance he had consumed prior to overdose, still loaded with 0.2 ml of prepared “China White”. The sample tested positive for fentanyl and xylazine. The patient’s urine tested positive for fentanyl, xylazine and methamphetamine. The patient received counseling about the risks of xylazine when combined with fentanyl.

## Discussion

These three clinical cases highlight a range of typical scenarios of xylazine-involved overdose seen by the overdose response team at the Prevencasa community harm reduction clinic in Tijuana, Mexico. In the early months of 2024, due to the arrival of xylazine, a very notable shift in the clinical characteristics of overdose events was observed, which required the overdose response team to adapt its practices. Patients who would have previously quickly regained consciousness after a 4.0 mg dose of intranasal naloxone [16] began to exhibit 10–30-minute periods of unconsciousness, despite adequate oxygenation as measured with pulse oximetry. Patients increasingly began to awaken in a confused or delirious state, exhibiting aggression, and often would expose themselves to physically dangerous circumstances (e.g. walking in traffic, laying on roadways) appearing to be unaware of their surroundings, significantly complicating field management. During this time, routine urine toxicology was offered to patients, as was direct testing of substances and drug wrappers, all of which showed high rates of xylazine positivity, using several brands of lateral flow immunoassay testing strips [17] (Fig. 1, D-F).

In response, the clinical team developed a set of strategies to manage xylazine-involved overdoses (Table 1). One valuable lesson was the insight to carefully titrate—and at times even reduce or withhold entirely—the dose of naloxone provided to overdose patients. We hypothesized that local sales points decreased the concentration of fentanyl provided to consumers, replacing it with xylazine. Therefore, 4.0 mg intranasal doses of naloxone seemed to induce more problematic withdrawal symptoms in patients than prior to the arrival of xylazine, as seen in case 1. Affected individuals increasingly would awaken expressing concern about physically uncomfortable withdrawal symptoms, which often prompted an immediate search for repeat illicit opioid consumption, and complicated post-overdose clinical monitoring. Instead, the use of smaller intramuscular doses (such as in case 3), titrated to adequate oxygenation—not consciousness—provides more stable patient care and better outcomes. In urgent overdose response situations, there is a pressing temptation to administer additional naloxone to unconscious patients. However, the non-opioid sedation period induced by xylazine requires re-centering the focus on oxygenation and respiratory status, not consciousness. The team has therefore increased its use of field pulse oximetry as the key clinical parameter of interest, re-doubled efforts to focus on field airway management, including proper positioning, and the use of mobile oxygen tanks. In some instances, perhaps when xylazine is the predominant component driving sedation, adequate oxygenation status can be achieved without naloxone, and supplemental oxygen and airway management are the key techniques employed (such as in case 2 above). The assessment of respiratory status has therefore become a key priority for xylazine overdose management. Although community naloxone distribution has become common, and overdoses involving only opioids are often managed in practice by individuals who lack medical training, the prolonged period of sedation with potential airway compromise induced by xylazine may require specialized knowledge to manage. Community-based training in airway management has been suggested as a key response tactic to xylazine among harm reduction practitioners to solve this challenge [2]. 

An increased focus on scene safety is also needed. Xylazine induces a level of sedation that many consumers of illicit opioids are unprepared for [2]. During acute overdose events, scene management is paramount, including transporting patients to a safe area. The post-sedation delirium we have observed often requires careful and gentle redirection from the clinical team to ensure patients do not place themselves in harmful scenarios.

We have also provided xylazine testing strips to patients, and syringe exchange clients, and provided education to the local population about the risks of xylazine. Xylazine testing strips are a relatively new introduction to the market, and are currently approved for forensic purposes, including directly testing substances. However they have been demonstrated, in practice, to work on urine samples [18, 19]. One concern is that the thresholds used in commercially available xylazine strips may be too low, resulting in a risk of false negative results [20]. However, strips can also be given to participants to directly test their own drug supply before consumption. Many individuals seeking illicit opioids do not wish to consume xylazine—especially once they are aware of its associated health risks [2]. Providing participants with xylazine testing strips—coupled with adequate counseling from medical providers—can therefore empower them to shift their demand towards products with fewer health risks. This can also serve as a segue into a helpful conversation between patient and provider about harm reduction in the midst of active substance use, that can often lead to engagement in opioid substitution treatment.

Collectively these strategies have helped the clinical team manage xylazine-fentanyl overdoses in the field and educate the patient population about evolving health risks associated with shifts in the illicit drug supply. However, more research is needed about the clinical management of xylazine-involved overdoses, especially in community clinics relevant broadly to harm reduction contexts. The techniques we propose here should be validated with more formal clinical studies. Beyond the current response to xylazine-fentanyl combinations [2, 21], many of these techniques may also apply broadly to the evolving polysubstance crisis unfolding in North America [22], which increasingly also involves nitazenes, novel synthetic benzodiazepines, and other synthetic sedatives mixed with fentanyls. Across these drug categories, naloxone-resistant sedation and field airway management may be core issues.

**Table 1:** Strategies to manage xylazine-fentanyl overdose in the field

Strategy	Description
Field Pulse Oximetry	Portable, battery-powered pulse oximetry can be used in the field to monitor oxygenation status in patients who have not regained consciousness.
Titrate Naloxone to Oxygenation Status	Naloxone administration, either intramuscular or intranasal, can be titrated to oxygenation status, not level of consciousness, to avoid inducing unnecessary opioid withdrawal symptoms after the xylazine sedation period passes.
Portable Oxygen and Airway Management	Portable oxygen tanks, and proper positioning of patients (laying on their side, jaw thrust, visualize airway) can assist in improving oxygenation after naloxone administration, assess for vomit and gastric contents, employ portable suction as needed.
Scene Safety	Patients with prolonged periods of sedation are vulnerable to traffic, assault, and other physical risks. Response team staff members can help secure the scene.
Distribute Xylazine Testing Supplies	Testing strips for xylazine presence using immunoassay technology are inexpensive (about $1 per strip) and easy to use. They can be distributed to participants to assist them in screening their drugs. In practice many patients show positivity in urine samples, although the strips have not yet been FDA approved for this purpose, and false negatives may occur.
Communnity Education	Many patients prefer to avoid xylazine once they are aware of it, understand the health risks, and are equipped with testing strips to detect it. Providers can counsel patients about the risks of xylazine and suggest safer options. This can help encourage patients to switch to methadone or buprenorphine treatment.

## Data Availability

No datasets were generated or analysed during the current study.
